# Changes in the Major Odorants of Grape Juice during Manufacturing of Dornfelder Red Wine

**DOI:** 10.1021/acs.jafc.2c06234

**Published:** 2022-10-19

**Authors:** Stephanie Frank, Peter Schieberle

**Affiliations:** † Leibniz Institute for Food Systems Biology at the Technical University of Munich (Leibniz-LSB@TUM), Lise-Meitner-Straße 34, 85354 Freising, Germany; ‡ Fakultät für Chemie, Technische Universität München, Lichtenbergstraße 4, 85748 Garching, Germany

**Keywords:** Dornfelder grapes, Dornfelder red wine, barrique barrel, steel tank, stable isotopically substituted odorants

## Abstract

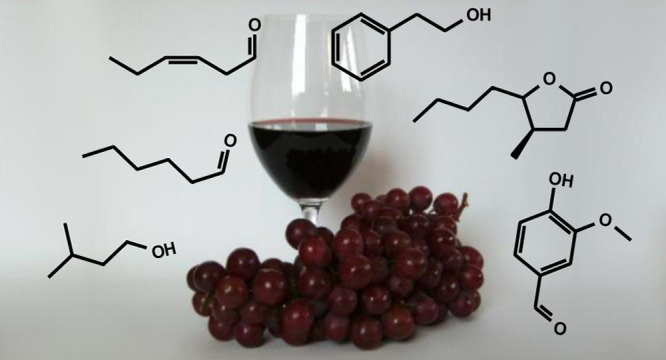

Application of the aroma extract dilution analysis (AEDA) on a distillate prepared from freshly squeezed juice of Dornfelder grapes revealed (3*Z*)-hex-3-enal and *trans*-4,5-epoxy-(2*E*)-dec-2-enal with the highest flavor dilution (FD) factors. In contrast, in the final Dornfelder wine prepared thereof, the highest FD factors were found for 2-phenylethyl acetate, 2-phenylethan-1-ol, and (*E*)-β-damascenone. However, for example, among others, (3*Z*)-hex-3-enal no longer appeared as an important odorant. To monitor the olfactory changes occurring in single processing steps from Dornfelder grapes to the final wine, selected odorants in grape juice, must, and young as well as aged wine from the same batch of Dornfelder grapes were quantitated. In particular, (3*Z*)-hex-3-enal and hexanal decreased considerably during mashing, while, as to be expected, the concentrations of yeast metabolites, e.g., odor-active alcohols and esters, drastically increased during fermentation. To reveal the influence of barrel aging, the odorants of the same Dornfelder wine aged in either barrique barrels or steel tanks were compared.

## Introduction

Besides color and taste, aroma is undoubtedly the most important quality attribute of wine, and thus, the identification of odorants has been a research topic in numerous investigations in the past, as documented in a selection of literature data.^1−7^ The results showed that, in particular, the grape variety, the fermentation process, and the storage in barrels are key parameters influencing the overall aroma of the final wine.

Several studies have already been undertaken to clarify the odorants of different grape varieties. Beak et al.^8^ and Fan et al.^9^ analyzed the odorants in grapes of the varieties Muscadine,^8^ Cabernet Gernischt,^9^ Cabernet Sauvignon,^9^ Cabernet Franc,^9^ and Merlot^9^ by gas chromatography–olfactometry (GC–O). Unexpectedly, Fan et al.^9^ identified the same odor-active compounds in all examined grape varieties, but their concentrations varied, thereby indicating that the characteristic odors of the different varietals depended upon quantitative rather than qualitative differences in the odor-active compounds.

In particular, the well-known amino acid metabolism, known as the Ehrlich degradation, leads to the formation of a certain group of potent odorants in many alcoholic beverages, such as wine, e.g., alcohols, aldehydes, and esters.^4,10,11^ Hernández-Orte et al.^12^ added selected amino acids to grape juice, for example, phenylalanine, and observed that the content of 2-phenylethan-1-ol was higher in the fermented grape juice supplemented with the amino acid than in the grape juice without the addition. Besides the amino acid metabolism, further odorants present in the final wine were either transferred directly from the grape juice or were formed from odorless precursors in grapes, such as glycosides and *S*-conjugates. The hydrolysis of glycosides can occur either enzymatically during fermentation or by acid hydrolysis during aging. Ugliano and Moio^13^ found that the yeast-driven enzymatic hydrolysis of glycosides was the major formation pathway for linalool and geraniol, while an acid hydrolysis led, among other compounds, to the generation of terpinen-4-ol and (*E*)-β-damascenone. In a last step of winemaking, storage in oak barrels has a huge influence on the aroma of the wine,^4^ and wines stored in oak, especially in barrique barrels, are commonly rated by the consumer to be of higher quality. For example, Jarauta et al.^14^ compared the volatiles of red wine that was stored in oak barrels to the same wine stored in stainless-steel tanks. Aging in oak affected many volatiles, including (4*R*,5*R*)-5-butyl-4-methyloxolan-2-one (whiskey lactone and oak lactone) and 4-hydroxy-3-methoxybenzaldehyde (vanillin), which are considered as key oak-derived compounds.^4^

To summarize, numerous studies have been published on the odor-active compounds of red wine, and also, the influence of single manufacturing steps on changes in wine volatiles has been reported. However, to the best of our knowledge, no data are available on changes of important odorants in Dornfelder grape juice on the way from grape juice to the final wine by application of the sensomics concept for the identification of odorants.^15^ Most previous studies were focused on either one single step of the manufacturing process or only a few odorants. Therefore, the aim of the present study was, first, to characterize the odorants in a freshly squeezed Dornfelder grape juice and in a steel tank-aged wine produced thereof. Second, monitoring the concentration of selected odorants during wine production using the same batch of grapes should be performed, and finally, major odorants in red wine of the same vintage and vineyard either stored in barrique barrels and steel tanks should be compared to elucidate the influence of the oak material on the odorant spectrum.

## Materials and Methods

### Samples

Dornfelder samples were obtained from a wine grower in the Rheinhessen region (Germany). Grape juice odorants were analyzed between 1 and 4 days after harvest of the fruits. For mash preparation, grapes were pressed, the mash was kept for 24 h, and a non-yeasted must was received after pressing. For wine preparation, *Saccharomyces cerevisiae* yeast was added to the non-pasteurized mash, and the material was fermented for 2 weeks. The fermented mash was pressed to obtain a young wine, of which one half was directly analyzed. The second half was filled in used French oak barrels and stored for 7 months. Another batch of young Dornfelder wine from the same grapes was stored in either steel tanks for 6 months or barrique barrels for 17 months. The barrique barrels (225 L) were made of French oak, and the barrels had already been used twice. Storage took place in a dark cellar at an average of 12 °C.

### Reference Odorants

Synthetic reference compounds for odorants **2**–**8**, **10**–**28**, **30**–**32**, **34**, **36**–**40**, and **42**–**44** were obtained from Merck (Darmstadt, Germany); compounds **9** and **35** were purchased from Lancaster (Mühlheim, Germany); compound **29** was a gift from Symrise (Holzminden, Germany); compound **41** was a gift from the Australian Wine Research Institute (AWRI, Adelaide, Australia); and compound **33** was synthesized as detailed in the literature.^16^

### Stable Isotopically Substituted Odorants

The synthesis of several internal standards was performed as recently described.^17^ In addition, the following compounds were synthesized as detailed in the literature: (^2^H_4_)-**8**,^18^ (^2^H_2_)-pent-1-en-3-one,^19^ (^2^H_2_)-**10**^20^ using a modified Lindlar catalyst,^21^ (^2^H_2–4_)-**14**^18^ using oct-3-yn-1-ol (Merck) as an educt, (^2^H_3_)-**15**,^22^ (^2^H_2_)-**20**,^23^ (^2^H_2_)-**21**,^24^ (^2^H_3_)-**25**,^25^ (^2^H_4_)-**33**,^26^ and (^2^H_4_)-**39**.^27^

### Gas Chromatography–Olfactometry (GC–O)

GC–O was performed using a Carlo Erba gas chromatograph type 5160 Mega series (Milano, Italy). The fused silica columns used were either an Agilent DB-FFAP or Agilent DB-5 column, both 30 m × 0.32 mm inner diameter, 0.25 μm film (Waldbronn, Germany). The initial oven temperature of 40 °C was held for 2 min, followed by a gradient of 6 °C/min. The final temperature of 230 °C (DB-FFAP) or 250 °C (DB-5) was held for 5 min. The injection volume was 1 μL. For GC–O, the effluent was split 1:1 by volume at the end of the column by means of a Y-shaped glass splitter and two deactivated fused silica capillaries (50 cm × 0.25 mm inner diameter). One half was conveyed to a flame ionization detector (FID) held at 240 °C, and the other half was conveyed to a heated sniffing port (250 °C). The method was performed as previously described.^17^

### Gas Chromatography–Mass Spectrometry (GC–MS)

For compound identification, 0.5 μL of the distillate was analyzed by means of a Hewlett-Packard 5890 Series II gas chromatograph (Heilbronn, Germany) connected to a Finnigan MAT 95 sector field mass spectrometer (Bremen, Germany). The fused silica columns used were either an Agilent DB-FFAP or Agilent DB-5 column, both 30 m × 0.25 mm inner diameter, 0.25 μm film. The oven temperature program was comparable to the GC–O analyses. Mass spectra were generated in the electron ionization (MS–EI) mode at 70 eV with a scan range of *m*/*z* 35–300. The Thermo Scientific Xcalibur software (Dreieich, Germany) was used for the evaluation of the mass spectra.

For compound quantitation, either a one-dimensional GC–MS system or a two-dimensional heart-cut GC–GC–MS system was used. As a one-dimensional instrument, a Varian CP 3800 gas chromatograph (Darmstadt, Germany) equipped with a CTC Analytics Combi PAL autosampler (Zwingen, Switzerland) was connected to a Varian Saturn 2000 mass spectrometer operated in the chemical ionization (MS–CI) mode with methanol as the reagent gas. The Agilent DB-FFAP column, 30 m × 0.25 mm inner diameter, 0.25 μm film, was operated as described above. The injection volume was 2 μL. The Varian MS Workstation software was used for the evaluation of the mass spectra. As a two-dimensional heart-cut instrument, a GC–GC–MS system with a Thermo Trace GC Ultra gas chromatograph equipped with a CTC Analytics Combi PAL autosampler was coupled to a Varian CP 3800 as the second gas chromatograph. The fused silica column in the first gas chromatograph was the Agilent DB-FFAP column, 30 m × 0.32 mm inner diameter, 0.25 μm film, as described above, and an Agilent DB-1701 column, 30 m × 0.25 mm inner diameter, 0.25 μm film, was installed in the second gas chromatograph. The column end in the first gas chromatograph was connected to a Thermo moving column stream switching (MCSS) device, and the column end in the second gas chromatograph was connected to a Varian Saturn 2200 mass spectrometer operated in the MS–CI mode with methanol as the reagent gas. The oven temperature programs were comparable, as mentioned above. The injection volume was 2 μL. The Varian MS Workstation software was used for the evaluation of the mass spectra.

### Isolation of Volatiles

The grape juice was obtained by means of a Philips Viva Collection, HR 1832/00 kitchen squeezer (Hamburg, Germany). Immediately after squeezing, an aqueous saturated calcium chloride solution (100 mL) was added to avoid enzymatic reactions. Volatiles were isolated by extraction with diethyl ether followed by application of the solvent-assisted flavor evaporation (SAFE).^28^ The distillate was concentrated to 1 mL. The detailed workup procedure applied to all samples was performed as previously described for red wine.^17^

### Aroma Extract Dilution Analysis (AEDA)

The concentrated volatile fractions were stepwise-diluted 1:2 with diethyl ether, and each diluted sample was subjected to GC–O. Each odorant was assigned a flavor dilution (FD) factor, representing the dilution factor of the highest diluted sample in which the odorant was detected during GC–O analysis. The analysis was carried out as previously described.^17^

### Odorant Quantitation

Various amounts of the respective sample (0.05–500 mL) were used depending upon the amounts of the target compounds estimated in preliminary experiments. The samples were spiked with defined amounts of the stable isotopically substituted odorants (resulting in concentrations of 1–5 μg/mL of each compound in the extract). After equilibration for 30 min, the volatiles and internal standards were extracted with diethyl ether and isolated by SAFE,^28^ as described above. Compounds were analyzed using either the one-dimensional GC–MS system (**11**, **16**, **22**–**24**, **31**, and **42**) or the heart-cut GC–GC–MS system (**2**, **4**–**5**, **7**–**10**, **13**–**15**, **17**, **20**–**21**, **25**, **28**–**30**, **32**–**36**, **38**–**40**, and **43**–**44**).

Peak areas of the analytes (**8**–**10**, **14**–**15**, **20**–**21**, **25**, **33**, and **39**) and the respective internal standards were calculated from the extracted ion chromatograms using the quantifier ions detailed in Table 1. The concentration of each target compound was then calculated from the area counts of the analyte peak, the area counts of the standard peak, the amount of Dornfelder sample used, and the amount of standard added, by employing a calibration line equation (Table 1). To obtain the calibration line equation, solutions of the reference analyte and standard were mixed in different concentration ratios and analyzed under the same conditions followed by linear regression. Detailed information, e.g., on quantifier ions, for compounds **2**, **4**–**5**, **7**, **11**, **13**, **16**–**17**, **22**–**24**, **28**–**32**, **34**–**36**, **38**, **40**, and **42**–**44** are given in the previous publication.^17^

**Table 1:** Internal Standards, Quantifier Ions, and Calibration Lines Used for the Quantitation of Selected Odorants

		quantifier ions (*m*/*z*)			
odorant	standard	analyte	standard	calibration line equation	*R*^2^
**8**	(^2^H_4_)-**8**	101	105	*y* = 1.0684*x* – 0.0831	0.999
**9**	(^2^H_2_)-pent-1-en-3-one	99	87	*y* = 0.6232*x* + 0.0171	1.000
**10**	(^2^H_2_)-**10**	81	83	*y* = 1.0143*x* – 0.0385	1.000
**14**	(^2^H_2–4_)-**14**	129	131–133	*y* = 1.1183*x* – 0.0306	1.000
**15**	(^2^H_3_)-**15**	153	156	*y* = 0.9712*x* – 0.0815	0.998
**20**	(^2^H_2_)-**20**	141	143	*y* = 1.1087*x* – 0.2196	0.997
**21**	(^2^H_2_)-**21**	137	139	*y* = 1.0381*x* – 0.1298	0.999
**25**	(^2^H_3_)-**25**	89	92	*y* = 0.9595*x* + 0.2309	0.999
**33**	(^2^H_4_)-**33**	139	143	*y* = 0.9294*x* + 0.0968	1.000
**39**	(^2^H_4_)-**39**	123	127	*y* = 0.7666*x* + 0.0060	1.000

Compound **1** was quantitated enzymatically using an ultraviolet (UV) test kit (R-Biopharm, Darmstadt, Germany).

## Results and Discussion

### Important Odorants in a Dornfelder Grape Juice

The distillate of Dornfelder grape juice obtained by solvent extraction and SAFE distillation^28^ was submitted to an AEDA, which allowed for the localization of 22 odor-active compounds with FD factors ranging from 16 to ≥8192 (Table 2). Preliminary structural assignments were achieved by comparing the linear retention indices (RIs) and odor descriptions of the odorants recorded during AEDA to published data compiled in the Leibniz-LSB@TUM odorant database.^29^ Structure proposals were then confirmed by analyzing the corresponding authentic reference compounds in an appropriate dilution by GC–O and GC–MS. The approach allowed for the structural assignment of all odor-active compounds (Table 2). High FD factors were determined for green, grassy smelling (3*Z*)-hex-3-enal (**10**) and metallic smelling *trans*-4,5-epoxy-(2*E*)-dec-2-enal (**33**). (3*Z*)-Hex-3-enal has already been reported as volatile in other grape varieties,^30^ whereas to our knowledge, *trans*-4,5-epoxy-(2*E*)-dec-2-enal has not been mentioned in grape juice before. With somewhat lower FD factors, hex-1-en-3-one (**9**; pungent), 4-hydroxy-3-methoxybenzaldehyde (**44**; vanilla-like), 3-isopropyl-2-methoxypyrazine (**15**; pea-like, earthy), and (*E*)-β-damascenone (**29**; cooked apple-like) were identified. Ten further odorants were detected with FD factors ranging from 512 to 128: acetic acid (**16**; vinegar-like), hexanal (**8**; green, grassy), (2*E*)-non-2-enal (**20**; fatty), 2-methoxyphenol (**30**; smoky), 3-*sec*-butyl-2-methoxypyrazine (**18**; bell pepper-like, earthy), 3-isobutyl-2-methoxypyrazine (**19**; green bell pepper-like, earthy), linalool (**21**; floral), (*R*)-carvone (**26**, mint-like), 4-hydroxy-2,5-dimethylfuran-3(2*H*)-one (**36**; caramel-like), and phenylacetic acid (**43**; honey-like). The entire results of the identification are summarized in Table 2. Apart from *trans*-4,5-epoxy-(2*E*)-dec-2-enal and hex-1-en-3-one, all identified compounds have already been mentioned in other grape varieties.^8,9,30−34^ However, the odor contributions of these volatiles were only partially confirmed in previous studies.

**Table 2:** Twenty Two Important Odorants (FD Factor of ≥16) in the Volatile Fraction of Dornfelder Grape Juice

number^a^	odorant^b^	odor^c^	RI^d^ DB-FFAP	FD factor^e^
**2**	ethyl 2-methylpropanoate	fruity	968	32
**8**	hexanal	green, grassy	1087	256
**9**	hex-1-en-3-one	pungent	1106	4096
**10**	(3*Z*)-hex-3-enal	green, grassy	1139	≥8192
**12**	(2*E*)-hex-2-enal	green apple	1214	16
**14**	octanal	citrusy	1279	32
**15**	3-isopropyl-2-methoxypyrazine	pea, earthy	1430	2048
**16**	acetic acid	vinegar	1445	512
**18**	3-*sec*-butyl-2-methoxypyrazine	bell pepper, earthy	1496	128
**19**	3-isobutyl-2-methoxypyrazine	green bell pepper, earthy	1518	128
**20**	(2*E*)-non-2-enal	fatty	1527	256
**21**	linalool	floral	1545	128
**26**	(*R*)-carvone	mint	1714	128
**29**	(*E*)-β-damascenone	cooked apple	1809	1024
**30**	2-methoxyphenol	smoky	1864	256
**31**	2-phenylethan-1-ol	floral, honey	1909	16
**33**	*trans*-4,5-epoxy-(2*E*)-dec-2-enal	metallic	2004	≥8192
**36**	4-hydroxy-2,5-dimethylfuran-3(2*H*)-one	caramel	2042	128
**38**	4-allyl-2-methoxyphenol^f^	clove	2167	16
**41**	rotundone^f^	pepper	2256	32
**43**	phenylacetic acid	honey	2570	128
**44**	4-hydroxy-3-methoxybenzaldehyde	vanilla	2575	4096

a All odorants were consecutively numbered according to their retention time on the DB-FFAP column.

b Each odorant was identified by comparing its retention indices on two fused silica columns of different polarity (DB-FFAP and DB-5), its mass spectrum obtained by GC–MS, as well as its odor quality perceived during GC–O to data obtained from authentic reference compounds analyzed under equal conditions.

c Odor quality as perceived at the sniffing port during GC–O.

d Retention index: calculated from the retention time of the compound and the retention times of adjacent *n*-alkanes by linear interpolation.

e Flavor dilution factor: dilution factor of the highest diluted sample prepared from the concentrated volatile fraction in which the odorant was detected during GC–O by three panelists.

f An unequivocal mass spectrum of the compound could not be obtained; identification was based on the remaining criteria detailed in footnote b.

To obtain deeper insight into the role of single odorants in the overall olfactory profile, odorants with high FD factors (at least from FD of 64) were selected for quantitation using one- or two-dimensional GC–MS systems. Stable isotopically substituted odorants were employed as internal standards. The results (Table 3) revealed concentrations ranging between very low concentrations of 0.087 μg/L for hex-1-en-3-one and high concentrations of 880 μg/L for hexanal, showing the highest amounts. The second highest concentration was determined for the second green, grassy smelling aldehyde (3*Z*)-hex-3-enal, followed by linalool and 4-hydroxy-3-methoxybenzaldehyde.

**Table 3:** Concentrations, OTCs, and OAVs of 12 Major Odorants in Dornfelder Grape Juice

number^a^	odorant	concentration^b^ (μg/L)	OTC^c^ (μg/kg)	OAV^d^
**10**	(3*Z*)-hex-3-enal^e^	91	0.12	760
**29**	(*E*)-β-damascenone	3.4	0.0060	570
**8**	hexanal	880	2.4	370
**2**	ethyl 2-methylpropanoate	30	0.089	340
**9**	hex-1-en-3-one	0.087	0.00069	130
**21**	linalool^f^	71	0.58	120
**15**	3-isopropyl-2-methoxypyrazine	0.28	0.0039	72
**20**	(2*E*)-non-2-enal	4.4	0.19	23
**14**	octanal	16	3.4	4.7
**33**	*trans*-4,5-epoxy-(2*E*)-dec-2-enal	0.59	0.22	2.7
**30**	2-methoxyphenol	1.5	0.84	1.8
**44**	4-hydroxy-3-methoxybenzaldehyde	65	53	1.2

a All odorants were consecutively numbered according to their retention time on the DB-FFAP column.

b Means of 2–3 repetitions; standard deviations were ≤15%.

c Orthonasal odor threshold concentration in water according to the Leibniz-LSB@TUM odorant database.^29^

d The odor activity value was calculated as ratio of the concentration to the odor threshold concentration.

e Concentration refers to a Dornfelder grape juice of the consecutive year.

f Odor threshold concentration of the racemate.

To assess the odor potency of the individual juice odorants, odor activity values (OAVs) were then calculated. Orthonasal odor threshold concentrations (OTCs) in the grape juice were approximated using OTCs in water. For 12 of the quantitated odor-active compounds, an OAV of above 1 was calculated; only these odorants are listed in Table 3. Green, grassy smelling (3*Z*)-hex-3-enal showed the highest OAV, followed by cooked apple-like smelling (*E*)-β-damascenone. Further OAVs of >100 were found for hexanal, fruity smelling ethyl 2-methylpropanoate, hex-1-en-3-one, and linalool. The high OAVs of green, grassy smelling aldehydes (3*Z*)-hex-3-enal and hexanal fully correlate with results of an olfactory profile analysis of the grape juice, with the green, grassy attribute predominating (data not shown). Both compounds are well-known constituents of fruits and leafy vegetables and are known to be formed by enzymatic reactions from linolenic and linoleic acids, respectively.^35^

### Identification of Important Odorants in a Dornfelder Red Wine Stored in Steel Tanks

The volatile fraction of a Dornfelder wine aged in steel tanks was isolated by solvent extraction and SAFE distillation.^28^ Application of the AEDA resulted in 30 odor-active compounds with FD factors ranging from 16 to ≥8192 (Table 4). Structural assignments were achieved as described above for the juice and allowed for the identification of all odor-active compounds (Table 4). High FD factors were determined for floral, honey-like smelling 2-phenylethyl acetate (**28**) and 2-phenylethan-1-ol (**31**), for cooked apple-like smelling (*E*)-β-damascenone (**29**), for malty smelling alcohols 2- and 3-methylbutan-1-ol (**11**), and for seasoning-like smelling 3-hydroxy-4,5-dimethylfuran-2(5*H*)-one (**40**). With somewhat lower FD factors, ethyl 2-methylpropanoate (**2**; fruity), ethyl 2-methylbutanoate (**5**; fruity), 3-isopropyl-2-methoxypyrazine (**15**; pea-like, earthy), and 4-allyl-2-methoxyphenol (**38**; clove-like) were identified. Seven further odorants were detected with FD factors ranging from 512 to 128: butane-2,3-dione (**3**; buttery), ethyl butanoate (**4**; fruity), 2- and 3-methylbutanoic acid (**24**; sweaty), phenylacetic acid (**43**; honey-like), butanoic acid (**23**; sweaty, cheesy), 5-pentyloxolan-2-one (**34**; coconut-like), and 4-hydroxy-2,5-dimethylfuran-3(2*H*)-one (**36**; caramel-like) (Table 4). The number of identified odorants was smaller in the steel tank-aged wine compared to the barrel-aged wine analyzed in our previous publication on Dornfelder wine,^17^ but most of the compounds were identical. This aspect will be discussed below.

**Table 4:** Important Odorants (FD Factor of ≥16) in the Volatile Fraction of Dornfelder Wine Aged in Steel Tanks

number^a^	odorant^b^	odor^c^	RI^d^ DB-FFAP	FD factor^e^
**2**	ethyl 2-methylpropanoate	fruity	968	2048
**3**	butane-2,3-dione	butter	989	512
**4**	ethyl butanoate	fruity	1039	512
**5**	ethyl 2-methylbutanoate	fruity	1054	1024
**6**	pentane-2,3-dione	butter	1057	16
**7**	ethyl 3-methylbutanoate	fruity	1072	64
**11**	2- and 3-methylbutan-1-ol^f^	malty	1213	4096
**13**	ethyl hexanoate	fruity	1237	64
**15**	3-isopropyl-2-methoxypyrazine^g^	pea, earthy	1430	1024
**16**	acetic acid	vinegar	1445	64
**17**	3-(methylsulfanyl)propanal	cooked potato	1456	64
**19**	3-isobutyl-2-methoxypyrazine^g^	green bell pepper, earthy	1518	64
**21**	linalool	floral	1545	16
**22**	2-methylpropanoic acid	sweaty, cheesy	1565	16
**23**	butanoic acid	sweaty, cheesy	1627	256
**24**	2- and 3-methylbutanoic acid^f^	sweaty	1666	512
**25**	3-(methylsulfanyl)propan-1-ol	cooked potato	1714	64
**27**	pentanoic acid	sweaty	1734	32
**28**	2-phenylethyl acetate	floral, honey	1809	≥8192
**29**	(*E*)-β-damascenone	cooked apple	1809	≥8192
**30**	2-methoxyphenol	smoky	1864	32
**31**	2-phenylethan-1-ol	floral, honey	1909	≥8192
**34**	5-pentyloxolan-2-one	coconut	2026	256
**36**	4-hydroxy-2,5-dimethylfuran-3(2*H*)-one	caramel	2042	128
**37**	2-ethyl-4-hydroxy-5-methylfuran-3(2*H*)-one^g^	caramel	2082	16
**38**	4-allyl-2-methoxyphenol	clove	2167	1024
**40**	3-hydroxy-4,5-dimethylfuran-2(5*H*)-one	seasoning	2211	4096
**42**	decanoic acid	soapy	2273	16
**43**	phenylacetic acid	honey	2570	512
**44**	4-hydroxy-3-methoxybenzaldehyde	vanilla	2575	16

a All odorants were consecutively numbered according to their retention time on the DB-FFAP column.

b Each odorant was identified by comparing its retention indices on two fused silica columns of different polarity (DB-FFAP and DB-5), its mass spectrum obtained by GC–MS, as well as its odor quality perceived during GC–O to data obtained from authentic reference compounds analyzed under equal conditions.

c Odor quality as perceived at the sniffing port during GC–O.

d Retention index: calculated from the retention time of the compound and the retention times of adjacent *n*-alkanes by linear interpolation.

e Flavor dilution factor: dilution factor of the highest diluted sample prepared from the concentrated volatile fraction in which the odorant was detected during GC–O by three panelists.

f These odorants were not separated on the fused silica column used for AEDA; the FD factor refers to the mixture.

g An unequivocal mass spectrum of the compound could not be obtained; identification was based on the remaining criteria detailed in footnote b.

### Changes in the Concentrations of Selected Odorants in Single Steps of the Manufacturing Process of a Dornfelder Wine

**Figure 1:**
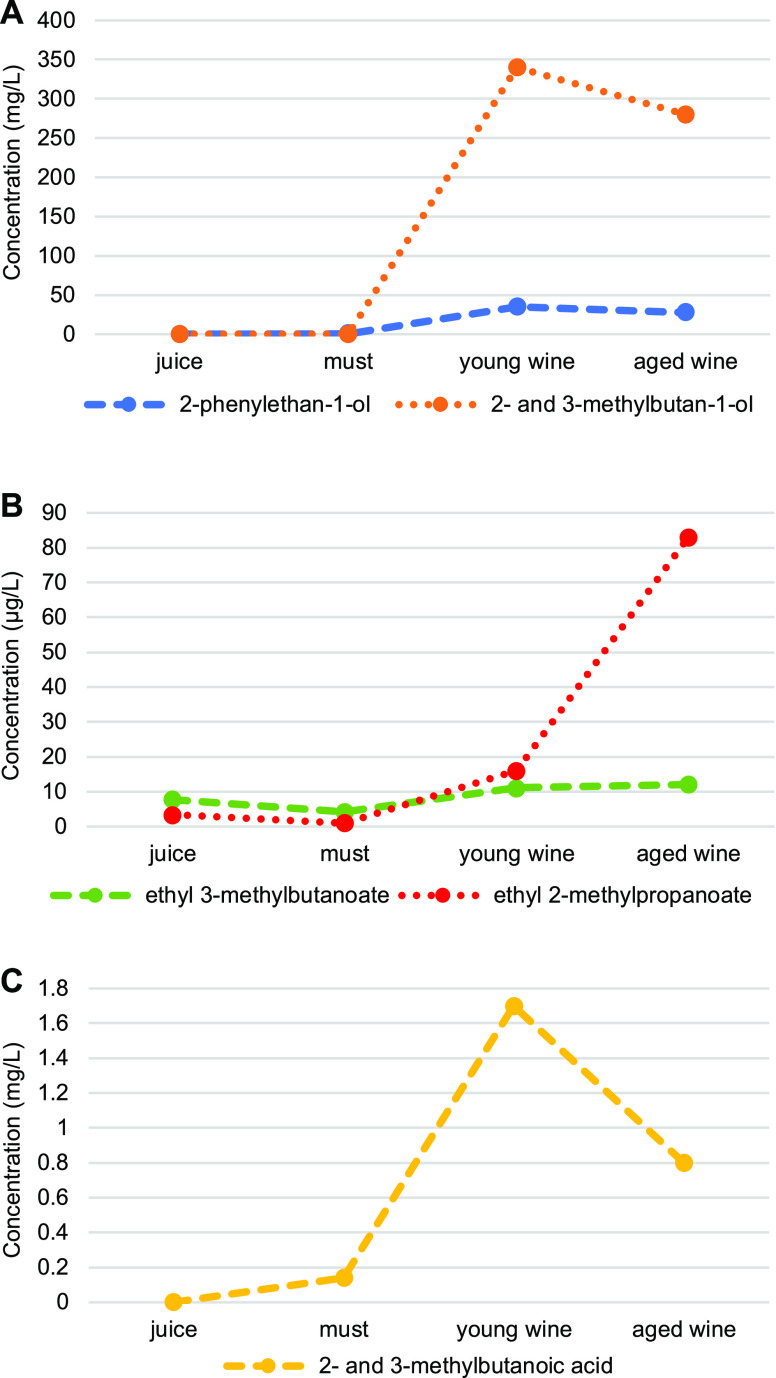
Changes in the concentrations of selected alcohols, ethyl esters, and acids during manufacturing of Dornfelder red wine. Concentrations are given in Table 5. Standard deviations were ≤12%.

A comparison of the odorants in the Dornfelder grape juice (Table 2) to those in the Dornfelder wine (Table 4) showed large differences. Therefore, to visualize the impact of single steps in the entire manufacturing process on the overall olfactory profile, selected odorants of different chemical classes were quantitated in a Dornfelder grape juice, must, and young and oak wood-aged wine, taken from the same batch of grapes. Volatiles from the individual samples were isolated and quantitated using stable isotopically substituted odorants as internal standards. The highest concentration in this batch of Dornfelder grape juice was detected for hexanal (1300 μg/L) (Table 5). The concentration of the second green, grassy smelling compound, (3*Z*)-hex-3-enal, was determined with 91 μg/L in the juice. Both aldehydes decreased during the manufacturing process, and while (3*Z*)-hex-3-enal was only detected in the juice, hexanal decreased by a factor of 20 during mashing but was no longer detectable in the wine. This was probably due to a reduction to hexan-1-ol during alcoholic fermentation.^36^ Besides hexanal and (3*Z*)-hex-3-enal, also the concentrations of linalool, ethyl 3-methylbutanoate, ethyl 2-methylpropanoate, 2-methoxyphenol, and (*E*)-β-damascenone decreased during mashing, probably as a result of bioconversions caused by grape enzymes (Table 5). By destruction of the grape cells during mashing, the enzymes present in the cells are released and an enhanced enzyme reaction is possible. For example, Oliveira et al.^37^ traced the decrease of C_6_-aldehydes to enzyme reactions, and Rapp et al.^38^ attributed the decrease of ethyl esters to enzymatic reactions during mashing. During fermentation, the concentrations of 2-phenylethan-1-ol, linalool, ethyl 3-methylbutanoate, 2-methoxyphenol, (*E*)-β-damascenone, 3-hydroxy-4,5-dimethylfuran-2(5*H*)-one, 2- and 3-methylbutanoic acid, 2- and 3-methylbutan-1-ol, but also ethyl 2-methylpropanoate increased (Table 5). 2-Phenylethan-1-ol and 2- and 3-methylbutan-1-ol are well-known compounds formed by yeast metabolism and are undoubtedly formed by a degradation of the respective parent amino acids 2-phenylalanine, isoleucine, and leucine following the Ehrlich pathway. Figure 1 shows the influence of the manufacturing process of Dornfelder wine on the concentrations of odorants formed by yeast fermentation. Except for ethyl 2-methylpropanoate, which was predominately formed during aging, juice fermentation was the main step in the formation of the odor-active alcohols, ethyl esters, and acids.

**Table 5:** Concentrations^a^ (μg/L) of Selected Odorants in Dornfelder Grape Juice, Must, and Young and Oak Wood-Aged Wine Taken from the Same Batch of Grapes

number^b^	odorant	juice	must	young wine	aged wine
**8**	hexanal	1300	66	nd^c^	nd^c^
**31**	2-phenylethan-1-ol	100	500	35000	28000
**10**	(3*Z*)-hex-3-enal	91	nd^c^	nd^c^	nd^c^
**21**	linalool	48	5.2	14	50
**44**	4-hydroxy-3-methoxybenzaldehyde	25	40	11	110
**7**	ethyl 3-methylbutanoate	7.8	4.2	11	12
**2**	ethyl 2-methylpropanoate	3.4	0.93	16	83
**30**	2-methoxyphenol	1.9	0.61	2.0	6.0
**29**	(*E*)-β-damascenone	1.7	<0.1	1.1	1.4
**40**	3-hydroxy-4,5-dimethylfuran-2(5*H*)-one	<0.1	0.18	1.0	2.5
**24**	2- and 3-methylbutanoic acid^d^	nd^c^	140	1700	800
**11**	2- and 3-methylbutan-1-ol^d^	nd^c^	nd^c^	340000	280000

a Means of 2–3 repetitions; standard deviations were ≤12%.

b All odorants were consecutively numbered according to their retention time on the DB-FFAP column.

c Not detected during GC–O analysis.

d These odorants were not separated on the fused silica column used for quantitation; the concentration refers to the mixture.

### Influence of Aging in Oak Barrels on Important Odorants in Dornfelder Red Wine

Dornfelder wine prepared from the same batch of grapes was aged in either barrique barrels or steel tanks, and 26 major odorants were quantitated (Table 6). The highest concentration was determined for acetic acid in barrique barrels (560 mg/L) compared to 270 mg/L in steel tanks. Similar data were found for 2- and 3-methylbutan-1-ol (350 mg/L, barrique; 290 mg/L, steel) and 2-phenylethan-1-ol (26 mg/L, barrique; 21 mg/L, steel). Nearly all quantitated odorants increased as a result of the oak wood contact. Only 2-phenylethyl acetate decreased. As to be expected, the only compound exclusively detected in the barrel aged wine was (4*R*,5*R*)-5-butyl-4-methyloxolan-2-one. Otsuka et al.^39^ identified 4-[(3,4-dihydroxy-5-methoxybenzoyl)oxy]-3-methyloctanoic acid in oak wood and suggested it as the precursor of 5-butyl-4-methyloxolan-2-one. Masson et al.^40^ later clarified the pathway for the formation of 5-butyl-4-methyloxolan-2-one. This component may occur in four stereoisomers, but in oak wood, only two of them were previously identified.^41^ According to Garde-Cerdán and Ancín-Azpilicueta,^42^ (4*R*,5*R*)-5-butyl-4-methyloxolan-2-one is regarded as the most important volatile of oak wood extracting into wine during barrel aging.

**Table 6:** Concentrations^a^ (μg/L) of Odorants in Dornfelder Red Wine Aged in Either Barrique Barrels (BBA) or Steel Tanks (STA)

number^b^	odorant	BBA	STA
**16**	acetic acid	560000	270000
**11**	2- and 3-methylbutan-1-ol^d^	350000	290000
**31**	2-phenylethan-1-ol	26000	21000
**1**	acetaldehyde	5000	3700
**25**	3-(methylsulfanyl)propan-1-ol	2500	95
**24**	2- and 3-methylbutanoic acid^d^	1500	1100
**22**	2-methylpropanoic acid	1400	1200
**23**	butanoic acid	1300	250
**13**	ethyl hexanoate	540	210
**42**	decanoic acid	450	190
**44**	4-hydroxy-3-methoxybenzaldehyde	260	8.6
**4**	ethyl butanoate	240	240
**39**	4-ethylphenol	92	3.1
**32**	(4*R*,5*R*)-5-butyl-4-methyloxolan-2-one	59	nd^c^
**43**	phenylacetic acid	51	32
**36**	4-hydroxy-2,5-dimethylfuran-3(2*H*)-one	19	9.6
**28**	2-phenylethyl acetate	16	86
**34**	5-pentyloxolan-2-one	15	12
**7**	ethyl 3-methylbutanoate	13	7.9
**38**	4-allyl-2-methoxyphenol	9.4	12
**5**	ethyl 2-methylbutanoate	7.6	6.6
**40**	3-hydroxy-4,5-dimethylfuran-2(5*H*)-one	7.4	1.1
**30**	2-methoxyphenol	6.9	3.1
**35**	4-ethyl-2-methoxyphenol	5.8	0.21
**17**	3-(methylsulfanyl)propanal	3.5	0.83
**29**	(*E*)-β-damascenone	2.7	3.2

a Means of 2–3 repetitions; standard deviations were ≤16%.

b All odorants were consecutively numbered according to their retention time on the DB-FFAP column.

c Not detected during GC–O analysis.

d These odorants were not separated on the fused silica column used for quantitation; the concentration refers to the mixture.

A further difference in the odorants between the steel tank and the barrique barrel-aged wine was, for example, 3-(methylsulfanyl)propan-1-ol, which increased by a factor of more than 25 in the barrel wine (Table 6). Also, the amounts of 4-hydroxy-3-methoxybenzaldehyde, 4-ethylphenol, and 4-ethyl-2-methoxyphenol increased nearly 30 times during barrel aging. The higher concentrations of these three compounds in the barrique wine are suggested to be a result of lignin degradation, because the ring substitution of ferulic acid is common in all compounds. The pyrolysis of lignin is well-known during barrel toasting. Spillman et al.^43^ confirmed that, in oak barrels, 4-hydroxy-3-methoxybenzaldehyde was formed as a lignin degradation product, mainly during coopering. Chatonnet et al.^44^ mentioned that 4-ethylphenol and 4-ethyl-2-methoxyphenol might have a microbiological origin. They supposed that these compounds were formed in wines during aging by some yeast species belonging to the genus *Brettanomyces* in the presence of hydroxycinnamic acid.

The following odorants only showed a slight increase during barrique aging: acetic acid, butanoic acid, ethyl hexanoate, decanoic acid, 4-hydroxy-2,5-dimethylfuran-3(2*H*)-one, 3-hydroxy-4,5-dimethylfuran-2(5*H*)-one, 2-methoxyphenol, and 3-(methylsulfanyl)propanal (Table 6). Contrary, the concentrations of 2- and 3-methylbutan-1-ol, 2-phenylethan-1-ol, 2-methylpropanoic acid, ethyl butanoate, 5-pentyloxolan-2-one, 4-allyl-2-methoxyphenol, ethyl 2-methylbutanoate, and (*E*)-β-damascenone were identical in wines prepared by both aging procedures (Table 6).

The study confirmed for the first time the molecular background of the huge olfactory changes occurring on the way from grape juice to the final Dornfelder red wine. Interestingly, of the selected quantitated odorants, only the concentrations of linalool and (*E*)-β-damascenone were identical in the grape juice and the final Dornfelder wine prepared thereof. However, the amounts of the free compounds present in the juice were degraded/lost during must preparation but were then released during fermentation/aging from their precursors in the juice. In general, the key steps in the formation of the overall olfactory profile of Dornfelder red wine are the significant reduction of the concentrations of both green, grassy smelling aldehydes delivered from the juice as well as the formation of yeast metabolites generated by amino acid degradation. Aging in a steel tank did not show differences in most of the major odorants of the Dornfelder red wine, but the steel tank wine consequently did not contain odorants released from the oak barrels, such as (4*R*,5*R*)-5-butyl-4-methyloxolan-2-one.
